# Supplementary material for: Reconciling Mining with the Conservation of Cave Biodiversity: A Quantitative Baseline to Help Establish Conservation Priorities
Source: PLoS One. 2016 Dec 20;11(12):e0168348. doi: 10.1371/journal.pone.0168348 (PMC5173368; doi:10.1371/journal.pone.0168348)
Supplement: S1 Dataset — (ZIP) [file pone.0168348.s002.zip › Taxa/Serra Norte/SN_2013/N5S/Lista N5S-03.pdf]

CAVIDADE N5S-0003

Classe	Ordem	Fam/Outros	Gên/Outros	Espécie	S-U	U-S	Zon.
Annelida	Oligochaeta			jovem	1		P
Arachnida	Acari	Cunaxidae		sp.1	X		P
Arachnida	Acari	Ixodidae		jovem	X		P
Arachnida	Acari	Trombidiformes		sp.49	X		P
Arachnida	Acari			jovem	X	X	
Arachnida	Amblypygi	Charinidae	*Charinus*	sp.1	1		E
Arachnida	Amblypygi	Phrynidae	*Heterophrynus*	*longicornis*		X	
Arachnida	Araneae	Caponiidae		sp.1	X	X	P
Arachnida	Araneae	Drymusidae	*Drymusa*	*spelunca*	X	X	E/P
Arachnida	Araneae	Ochyroceratidae	*Ochyrocera*	sp.1	X	X	E/P
Arachnida	Araneae	Ochyroceratidae	*Speocera*	sp.1	X		P
Arachnida	Araneae	Oonopidae	aff. *Xyccarph*	sp.2	X		P
Arachnida	Araneae	Pholcidae		jovem	X	X	P
Arachnida	Araneae	Salticidae		sp.7	X		E
Arachnida	Araneae	Scytodidae	*Scytodes*	jovem	1		E
Arachnida	Araneae	Theridiidae	*Theridion*	sp.		X	
Arachnida	Araneae	Theridiidae	*Theridion*	sp.1	X		E
Arachnida	Araneae	Theridiidae	*Theridion*	sp.4	X		P
Arachnida	Araneae	Theridiosomatidae	*Plato*	sp.1	X		E/P
Arachnida	Opiliones	Escadabiidae		sp.2	X	X	P
Arachnida	Palpigradi	Eukoeneniidae	*Eukoenenia*	sp.1	X		P
Arachnida	Pseudoscorpiones	Bochicidae		jovem	X		P
Arachnida	Pseudoscorpiones	Chthoniidae	*Pseudochthonius*	sp.1	X		E/P
Arachnida	Pseudoscorpiones	Olpiidae		sp.1	X		E
Chilopoda	Scolopendromorpha	Scolopocryptopidae	*Dinocryptops*	*miersii*	2		P
Chilopoda	Scutigeromorpha	Pselliodidae	*Sphendononema*	jovem	4		E/P
Diplopoda	Polydesmida	Pyrgodesmidae		jovem	X	X	P
Entognatha	Collembola	Entomobryidae		sp.3	X		E
Entognatha	Collembola	Paronellidae		sp.1	X		E
Entognatha	Collembola	Sminthuroidea		sp.2	X		P
Entognatha	Collembola			sp.		X	
Insecta	Blattodea			jovem	1	X	E
Insecta	Coleoptera	Carabidae		sp.3	X	X	P
Insecta	Coleoptera	Coccinellidae		sp.1	X		E
Insecta	Coleoptera	Staphylinidae	Goniacerini	sp.		X	
Insecta	Diptera	Chloropidae		sp.	X		P
Insecta	Diptera	Culicidae	*Culex*	sp.	X		P
Insecta	Diptera	Phoridae	Phorinae	sp.	X		P
Insecta	Diptera	Psychodidae	Phlebotominae	sp.	X		P
Insecta	Heteroptera	Dipsocoroidea		sp.	X		P
Insecta	Heteroptera	Reduviidae	Emesinae	jovem		X	
Insecta	Heteroptera	Reduviidae	Reduviinae	sp.	2		E
Insecta	Homoptera	Cixiidae		jovem	X	X	P
Insecta	Hymenoptera	Formicidae	*Dolichoderus*	aff. *bispinosus*	X		P
Insecta	Hymenoptera	Formicidae	*Eurhopalothrix*	sp.1	X		P
Insecta	Hymenoptera	Formicidae	*Gnamptogenys*	sp.2	X		P
Insecta	Hymenoptera	Formicidae	*Hypoponera*	sp.1	X		P
Insecta	Hymenoptera	Formicidae	*Paratrechina*	sp.		X	
Insecta	Hymenoptera	Formicidae	*Solenopsis*	sp.1	X		E

Insecta	Hymenoptera	Formicidae	*Trachymyrmex*	sp.1	X		P
Insecta	Isoptera	Termitidae	*Embiratermes*	sp.	X		E
Insecta	Lepidoptera	Noctuoidea		sp.3	1		P
Insecta	Lepidoptera	Noctuoidea		sp.5	1		P
Insecta	Lepidoptera	Tineoidea		jovem		X	
Insecta	Orthoptera	Phalangopsidae	*Phalangopsis*	sp.	34		P
Insecta	Psocoptera	Epipsocidae	*Epipsocus*	sp.2	X		P
Insecta	Thysanura	Nicoletiidae		sp.1	X	X	
Gastropoda	Pulmonata	Systrophiidae	*Happia*	sp.		X	
Malacostraca	Isopoda	Philosciidae		sp.1	X	X	P
Symphyla		Scutigerellidae		sp.		X	
Amphibia	Anura	Strabomantidae	*Pristimantis*	cf. *fenestratus*	3		E/P
Mammalia	Chiroptera	Emballonuridae	*Peropteryx*	sp.		X	
Mammalia	Chiroptera	Furipteridae	*Furipterus*	*horrens*		X	
Mammalia	Rodentia	Cricetidae	*Rhipidomys*	sp.		2	P
